# Ten simple rules for reporting a bug

**DOI:** 10.1371/journal.pcbi.1010540

**Published:** 2022-10-13

**Authors:** Benjamin C. Haller

**Affiliations:** Department of Computational Biology, Cornell University, Ithaca, New York, United States of America

## Introduction

I work on a software program called SLiM [1]. What it does is unimportant here; the important bit is that it’s open source on GitHub, it has perhaps a couple of hundred users, and those users report bugs (real or otherwise) to me as they use my software. Bugs, of course, are flaws in computer software—flaws that can make the software crash, become unresponsive, or produce incorrect output, or that make the software hard to use or understand. I’ve actually been a software developer for more than 40 years now, depending upon where you draw the line; before I switched to being a scientist, I worked at Apple, where I had the perhaps dubious distinction of having filed more bugs in their bug-reporting database, at that time, than any other person. This did not make me so many friends; the path of the avid bug reporter can be a lonely one. (*Caveat lector*!) The point is, I have quite a bit of experience on both ends of the process—both writing and reading bug reports—and I can tell you that it is a process that is plagued by difficulties and annoyances on both ends. Here, then, are 10 simple rules for reporting a bug. If you follow them, the bug you report will be more likely to get fixed, and fixed quickly. You will also make your friendly neighborhood software developer happy, which is, of course, foremost in your mind as you angrily type out a bug report about a stupid, annoying problem that is plaguing you. That brings us to the first rule!

## Rule 1: Be patient

Mostly, people are very nice, in the bug reports they file, but I’ve gotten a couple that would really raise your eyebrows—hostility, and blame, and nasty remarks about the software, and personal attacks. Don’t be that person. Maybe that’s obvious, but it’s important, so I’ll nevertheless explain a few reasons.

One is “enlightened self-interest.” A good way to pretty much guarantee that the bug you’re reporting will not get fixed promptly, if at all, is to attack the developer; the developer, understandably, will not feel much interest in helping you in return (although your bug might get fixed anyway if it’s critical—causing incorrect results to be produced, for example).

Many software developers, especially in science but also for other open-source software, are essentially volunteers. Many of them are literally coding in their free time; even if that is not the case, coding and support still often take them away from the things they are officially paid to do. This is not a reason not to file bugs—most developers care about their software and want it to be better, so they actually welcome bug reports. (I love getting a good bug report!) It’s just a reason to be nice.

Sometimes you might think “Wow, this is such a dumb, obvious bug—how can it not be fixed already?” Honestly, I think this sometimes too—sometimes even about bugs in my own software. You might conclude that the developer is lazy or incompetent. In reality, however, the bug you found might happen only with your specific usage pattern, or your specific input data, or only on your machine for some reason, or only with a particular version of other software (R, Python, the compiler …). *You* might hit the bug every time, but it is still possible that nobody else has ever seen the bug at all.

You might also discover, after further investigation, that the bug is actually on your end! Perhaps your input data is incorrect; perhaps you are using the software incorrectly; perhaps your understanding of what the software does is incorrect. Keep this possibility in mind as you file your report; save yourself having to apologize for an angry bug report when it turns out that the problem was your fault all along.

Keep in mind also that you are probably not the only user of the software. What is important to you might not be what is important to other users, and sometimes, software development involves triage. The bug that’s biting you (but nobody else) every time you use the software might be less important to fix than the bug that’s biting everybody else (but not you). Don’t let that stop you—the developer will appreciate catching an obscure bug before it bites other users.

Finally, keep in mind that people go on vacation, many people don’t check their email at all on weekends, and so forth. In my field of evolutionary biology, many people have a “field season” that can last several months, during which they are off in some remote part of the world that might lack internet access, or even electricity. People also get sick, have family emergencies, and so forth. If you need something fixed quickly, you can mention that in your report so the developer knows the bug is urgent, and you can reach out to others in the software’s community to see if someone can help. If the software is open source, you could even help out by fixing it yourself—see Rule 10.

## Rule 2: Check whether the bug is already fixed

Filing a bug that has already been fixed is a waste of time for you and for the developer. The first thing to ask yourself is therefore “Am I using the current version of this software?” If not, upgrade and try again to see whether the bug still bites you. A bug report filed against an old version of a program is not very useful and is unlikely to be a high priority. If you really do need to file a bug against an old version, be sure to mention that in your report, and explain why.

If the software is open source, you might even try the current “development version” of it to see whether the bug has been fixed there. This is more of a headache; you’ll need to build the software from its source code, rather than using an installer, and the current development version might not be in a very usable state—it might contain much worse bugs! That being said, many projects maintain a “development branch,” and most developers will be happy to help you get that up and running. I don’t expect users to try this, in general; but if you’re familiar with software development, or if you’re considering trying to find and fix the bug yourself, it might be a good idea.

## Rule 3: Check for a previous report

Similarly to Rule 2, it’s also useful to check whether the bug has already been filed by someone else. Open source software, such as software on GitHub, usually has a public issue database where you can look up bugs. If someone else has already filed the bug, save yourself the work!

Finding an existing bug can be helpful in other ways, too. A quick “me, too!” comment on a bug lets the developer know the issue is being hit by others. You might also find information about a workaround or a “band-aid” that will allow you to make progress while waiting for the bug to be fixed.

Also, don’t forget about the wonderful resource known as the internet. Try Google; maybe there is an answer out there, in some blog post, or perhaps on stackoverflow or a similar website. My software, SLiM, has an associated discussion group where issues get discussed and resolved; for my users, that’s a good place to look. When I hit an issue with some program, I usually find that many other people have hit it before me.

## Rule 4: Use the right bug-reporting channel

Different developers prefer to receive bug reports through different channels. You might check the documentation, or the website for the program. Most open-source projects have an established way to report and track bugs, whether via GitHub issues, a bug tracker such as BugZilla or JIRA, or posts on a discussion forum. Twitter, email, homing pigeon? If there is a bug-reporting template provided, use that, even if it seems annoying; the developer probably has a good reason for having set it up. If you go through the wrong channels, or don’t use the template provided, it will take the developers longer to identify and fix your issue.

## Rule 5: Describe the problem in detail

This is a really important one. Surprisingly often, I get a bug report that just says something like “I clicked the button and it didn’t work.” First of all, which button? And what did you do before that, and what input data had you provided, and so forth? This sort of confusion goes into the concept of the “minimal reproducible example,” which is Rule 6, so I’ll leave it for later. Second of all, though, what do you mean when you say that it “didn’t work”? Did the program hang, meaning that it became permanently unresponsive? (How long did you wait, in fact, before concluding that it had hung?) Did it crash? Did it work eventually, but take an unreasonably long time? Did it produce incorrect or unexpected output? No output at all? Did it exhibit visual glitches of some kind? Weird noises? Smoke?

One thing that can be very useful is a screenshot; “a picture is worth a thousand words,” as they say. It’s very common to have a long back-and-forth discussion about the details of a bug, and then, when I finally see a screenshot, realize that the problem is just a trivial misunderstanding of some sort.

Another thing that is often useful is the output from the software. Not just the actual error message, if an error was reported to you, but the output that came before that, too. Fixing a bug is often rather like detective work—finding clues and piecing together evidence. The complete output log from a program is often a treasure trove of clues and evidence that lead to the culprit.

Finally, sometimes a bug can really be a case of a mismatch between expectations and reality. This can manifest as a bug report that says “I clicked the button and it did X,” where my reaction, as the developer of the software, is “Yes, that is what that button does!” It is therefore often important to say what you *expected* to happen instead. Perhaps you misunderstood what the software does; perhaps the software does what you expect, but not in the way that you expect it to; perhaps your “bug report” is really a “feature request,” meaning that you have an idea for how the software could be *better* at what it does. The developer doesn’t know what you expected to happen, or wanted to happen, unless you tell them.

## Rule 6: Provide a minimal reproducible example

A developer can find and fix your bug much more easily if they can straightforwardly reproduce the problem on their own system, so that they can trace exactly what has gone wrong. Providing a minimal reproducible example [2] might therefore be the most important rule to follow when writing a bug report; unfortunately, few users do it, which creates big headaches for developers. If you remember one rule from this essay, please remember this one.

What is a minimal reproducible example? Let’s tackle that phrase from end to beginning. An “example,” for a bug report, is a set of instructions that made the bug happen. Maybe only once; maybe you didn’t even try a second time. (When the bug erased all your files, it made you a bit wary of trying again!) An example is a small clue to the developer regarding what might have gone wrong, but only a small clue.

A “reproducible example” is a set of instructions that makes the bug happen every time (ideally), or at least often. You’ve tried following the steps in the example several times, and you’ve seen the bug more than once. For an example to be “reproducible,” it needs to be complete: You need to supply all of the input files necessary, all of the command-line options used, the random number generator seed that was used, and so forth. (Occasionally, confidentiality concerns may prevent you from sharing some materials in a public forum like a bug database; if so, some bug databases provide a confidentiality option, or you might send those materials to the developer privately. If you need to keep your example private even from the developer of the software you are using, perhaps for legal or commercial reasons, then try to put together a different example that is not confidential.) The developer needs to be able to reproduce the bug on *their* machine; that’s the point of the whole exercise. That’s important because to find and fix a bug, it is often invaluable to be able to see the bug actually happen. This lets the developer see it “in the debugger,” where a “debugger” is a separate program that software developers use to investigate bugs. It also lets the developer confirm that the bug has actually been fixed, by trying the reproducible example again, post-fix, and seeing that the bug no longer bites.

But this is still not enough; what is needed is a “*minimal* reproducible example.” To see why, imagine receiving a bug report that says “When I try to load this 87.3 GB input file, and let the program run for 37 days, it then crashes.” That procedure might be reproducible, but it’s still not really useful. Realistically, the developer can’t run the program for 37 days waiting for the bug to happen, and trying to figure out what, specifically, about your 87.3 GB input file triggered the bug will be a nightmare. You know what previous runs of the software you have done that worked; and you perhaps know what might be different and special about this particular input file that might be causing the crash. Use that knowledge to improve your bug report.

This exercise of making a reproducible example “minimal” is a bit like the old joke, “How do you make a statue of an elephant?” “Get yourself the biggest block of granite you can find, and then chip away everything that doesn’t look like an elephant.” In this case, you want to chip away everything that is unrelated to the bug, until all you are left with is what is really necessary to cause the bug. Make your input file/data as small and simple as possible; but also, make the problem you are giving to the software as simplified as it can be. In the case of my software, which runs evolutionary biology simulations, there are many switches that can be flipped and knobs that can be turned to make things simpler. If you set the mutation rate to zero, so that new mutations never occur, does the bug still happen? If so, then set it to zero. If you set the recombination rate to zero, so that no meiotic recombination scrambles up the genetics in the simulation, does the bug still happen? If so, then set it to zero. And so on. The goal is to eliminate as many possible sources of causation as you can. What remains—that which cannot be removed—the elephant in the room, so to speak—is, one hopes, directly related to the reason that the bug occurs.

Mention these things in your report. If you say “I tried setting the mutation rate to zero, and the bug went away, so I left mutations on,” that is extremely important and useful information. Similarly, if you can say “I was able to reproduce the bug in 4 out of 5 tries, following these steps,” that’s also very helpful; if I can’t make it happen on my side, following the same steps, then the reason for that discrepancy will be the first thing I investigate.

If you give me a minimal example that I can run in isolation, allowing me to see the bug in the debugger, I’m likely to fix it then and there—sometimes I post a bug fix within a few minutes of receiving a good reproducible report! (This sometimes startles people.) Otherwise, the bug is likely to go on the “back burner.” I will have to invest all the work of chipping away at the provided example to get something minimal that I can reproduce under the debugger, but without any of the knowledge and context possessed by the person who filed the report. Doing that can take hours or even days, and so it will wait until I have the time and energy.

## Rule 7: Provide all relevant information

There’s a lot of contextual information that is important to your bug report. What platform are you on—Linux, macOS, Windows? What version of that platform? What version of the software are you running, and if there are different ways to install it, how did you install it?

Even hardware details can sometimes be relevant. If it is a graphics glitch, tell me about your graphics setup (external monitor, high-resolution display, video card). If it’s a problem with file input or output, tell me about that—are you trying to write to a networked volume, or is it possible you’re running out of disk space, or are you on a computing cluster that might have weird file permissions rules for the /tmp temporary directory?

Other software on your machine matters too. If the software interfaces with something like R or Python, what version of that are you running, and what other packages do you have installed? Could other programs be taking up a bunch of memory, such that the bug is related to out-of-memory problems? Are you running under a weird software environment like VirtualBox that might be relevant?

Much of this information might be provided by a “system report” generated by the software, or by your operating system. Many bug templates will request such a system report and provide information about how one ought to be generated. Even if it is not requested, you might attach one if you know how to do so.

Rule 6 already talked about providing complete information about the problem, in the form of a minimal reproducible example. Don’t forget to also provide all relevant information about the output. It’s very common to get a bug report that says “After doing x, y, and z, I got an error.” What was the error message? Was this an error from the software itself, or an error displayed by the operating system? Was there any other output, before or after the error? All of this matters.

## Rule 8: Be concise

Now that I’ve told you about all of the many bits of information you ought to include in your bug report, my next piece of advice to you is: be concise. These are not contradictory pieces of advice. Think again of the elephant statue: You need to provide everything that looks like an elephant (your minimal reproducible example, your information about your software platform, and so forth), but you also need to carve away everything that does not look like an elephant. “Let me begin this bug report with a history of my scientific career that led to my development of the model I am running” is not a good way to start. Even “Let me begin by describing in great detail exactly what research problem I am trying to solve and why I am using your software to solve it” is not so good, probably. I hesitate to say that that second example is *never* good; sometimes, that sort of background information will allow the developer to say “Ah, you probably want to use the software in this other way” or even “Ah, you really want to use this other software package instead.” But try to cut to the chase without too much warm-up prose: What did you try to do with the software, what happened that you didn’t like, what did you want to happen instead, and how exactly can the developer easily reproduce this problem?

## Rule 9: Stay engaged

It’s important to stay engaged with your bug report after you’ve filed it. You might think you’ve said everything there is to be said, but very often, the developer will have further questions for you that you’ll need to answer before work can proceed. If you stay plugged in, the process will go much faster. It’s very frustrating to receive an incomplete bug report, ask a question to try to fill in the missing information, and then get nothing but crickets for weeks—or never get a reply at all. Often, this means the bug report has to be closed, because the issue is simply not well understood and cannot be reproduced.

Staying engaged is particularly important if you were unable to supply a minimal reproducible example with your report. In this case, the developer may be unable to get the bug to happen on their own machine, and so they will have to engage in a “remote debugging” session with you, in which they try a fix to the hypothesized source of the bug, and then have you try out that fix on your machine to see whether it resolved your issue. This can take numerous rounds of back-and-forth. Be patient and stick with it; the developer is doing their best, and it’s difficult for them too. This is why it’s so much better to provide a minimal reproducible example with your report, of course—if the problem can be reproduced without your help, then you usually don’t need to be involved in the rest of the process.

Sometimes it can even be useful to do a “screen sharing” session with the developer, in which they control your screen remotely so they can play around with the issue themselves. This is possible to do with Zoom now [3], although most people don’t realize it—it’s a very useful feature! Perhaps even better, the website Jitsi (https://meet.jit.si) is a free, open-source alternative that also supports screen sharing. If you’re comfortable with sharing your screen, you might suggest it to the developer.

## Rule 10: Think of yourself as part of the team

This perspective applies mainly to open-source software projects, but it can be helpful when reporting bugs for other software too. Open-source projects are often a group collaboration, with many contributors. Frequently, contributors to an open-source project are not contributing to the code of the software itself but are instead helping to document it, to test it, to publicize it, to answer questions from users, to think of new ideas that would improve it, and so forth. By filing a bug report on such a project, you are a contributor to the project too!

If you think of yourself in this light, you may realize there are ways in which you can be a more useful contributor than just dropping a bug report on someone else’s doorstep [4]. Putting in the work to produce a minimal reproducible example (see Rule 6) is one very important way to contribute to the project (and it makes it much more likely that your bug will get fixed). But being a contributor can go well beyond that. Once you’ve figured out the minimal conditions necessary to reproduce the bug, perhaps you have an idea of what might be going wrong in the software, in terms of its internal workings. You can express that hypothesis in your bug report—“I think maybe the origin of the bug is that the code is not including the last piece of data in its analysis; maybe this is an off-by-one error in a loop.” Even better, you might be able to look at the code for the software and actually find the bug. Even better than *that*, you might be able to fix the bug in the software’s source code, test that your fix works (using your minimal reproducible example), and submit your fix to the project to be merged in. The absolute best bug reports are the ones that come with a fix.

That might sound scary, if you’re not a software developer yourself, but actually, an excellent way to learn to code is to start out by making small contributions to open-source projects. A fix to a simple bug is often just a tiny tweak to the code, and even if you’re not entirely familiar with the language the software is written in, it might not take long before you can understand the basic logical structure of it. Even if your fix is not correct, you might point the developer to the right area of the code base, and you will get to see the final, correct fix and learn from it. Soon, you might find yourself submitting a fix that gets accepted, or even implementing a new feature—or you might find it rewarding to contribute more to the project in some other way, which is great too.

## Conclusions

In a sense, all of the above can be condensed down to a single “Golden Rule”: Write the bug report that you would want to receive, if you were the developer. Put yourself in the developer’s sandals and try, if you can, to give them what you anticipate they will need to know; or, at a minimum, try to be nice and helpful and thoughtful. This is particularly important because bug reports can feel kind of critical and even accusatory: You are literally telling someone that they made a mistake—maybe sort of a dumb mistake, in all honesty, although you are too polite to say so—and the mistake they made caused you annoyance and difficulty. That can be hard, on both ends! As with the peer review process, which can similarly feel critical and even accusatory, a little kindness goes a long way toward softening the tone of the interaction.

The right way to file a bug report is often discussed in the developer community, and the rules given here are representative of prevalent views in the community. For those who would like to read more, a couple of pieces can be recommended here [2,5,6], and a search engine will lead you to many more.

It’s also worth noting that developers have some responsibilities in this interaction too. If their software ends up being unmaintained and unsupported “abandonware,” the developer should mark it as such for everybody’s sake, so people know. Otherwise, developers should make a good-faith effort to maintain their software and fix bugs, provide good documentation, support their users, and work to build a kind and helpful community around their software. To that end, there are a couple of Ten Simple Rules papers that deserve a shout-out, such as those for “helping newcomers become contributors to open projects” [7], “documenting scientific software” [8], “making research software more robust” [9], “developing usable software in computational biology” [10], and “open development of scientific software” [11].

It’s really rewarding to be a part of the open software scientific community. I enjoy meeting and talking to users, I love talking about how my software could be improved, and I even (usually) enjoy receiving bug reports. We’re all in this together; let’s try to make each other’s lives easier when we can, by carving beautiful elephants for each other.
